# LncRNA PLAC2 down‐regulates RPL36 expression and blocks cell cycle progression in glioma through a mechanism involving STAT1

**DOI:** 10.1111/jcmm.13338

**Published:** 2017-09-18

**Authors:** Yan‐Wei Hu, Chun‐Min Kang, Jing‐Jing Zhao, Ying Nie, Lei Zheng, Hai‐Xia Li, Xin Li, Qian Wang, Yu‐Rong Qiu

**Affiliations:** ^1^ Laboratory Medicine Center Nanfang Hospital Southern Medical University Guangzhou Guangdong China; ^2^ Department of Anesthesiology Guangdong 999 Brain Hospital Guangzhou Guangdong China

**Keywords:** lncRNA PLAC2, RPL36, STAT1, cell cycle, glioma

## Abstract

Current glioma therapies allow *in situ* delivery of cytotoxic drugs to the tumour; however, gliomas show early recurrence due to their highly proliferative character. Long non‐coding (lnc)RNAs play critical roles in tumorigenesis by controlling cell proliferation and cycling. However, the mechanism of action of lncRNAs in glioma development remains unclear. Here, we report that the lncRNA PLAC2 induces cell cycle arrest by targeting ribosomal protein (RP)L36 in glioma. RPL36 promoted cell proliferation and G1/S cell cycle progression. Mass spectrometry analysis revealed that signal transducer and activator of transcription (STAT)1 interacted with both lncRNA PLAC2 and the *RPL36* promoter. We also found that the nucleus PLAC2 bind with STAT1 and interact with RPL36 promoters but the cytoplasmic lncRNA PLAC2 inhibited STAT1 nuclear transfer, thereby decreasing RP36 expression, inhibiting cell proliferation and inducing cell cycle arrest. These results provide evidence for a novel cell cycle regulatory network in glioma comprising the lncRNA PLAC2 along with STAT1 and RPL36 that can serve as a therapeutic target for glioma treatment.

## Introduction

Gliomas are the most frequent primary tumours in the brain, accounting for 50–60% of all brain malignancies 1. Gliomas include a variety of histologies including astrocytomas and glioblastomas, which are common, and oligodendrogliomas, mixed oligoastrocytomas and pilocytic astrocytoma, which are less so 2. Glioblastomas are the most lethal type of primary brain tumour; patients have a median survival of <12 months due to resistance to radiation and other treatments 3. Many aspects of gliomas have been extensively studied, including tumour suppressor gene mutations, aberrant protein expression and cancer stem cell identification 4. However, effective strategies that can decrease glioma incidence and associated mortality are lacking 5.

Long non‐coding (lnc)RNAs are non‐protein‐coding transcripts longer than 200 nucleotides that regulate gene expression by a variety of mechanisms, including control of transcription, posttranscriptional processing, genomic imprinting, chromatin modification and regulation of protein function and thus play important roles in many biological processes including development, cell growth and tumorigenesis 6, 7, 8. In cancer, aberrant expression of and mutations in genes encoding lncRNAs can contribute to tumour development and progression by regulating tumour cell proliferation, invasion, metastasis and survival 9, 10, 11. The discovery of dysregulated lncRNAs represents a new layer of complexity in the molecular architecture of human disease. However, there are still many gaps in our current understanding of lncRNA function 12. PLAC2 (ENSG00000223573) is a 3.7‐kb lncRNA locate in Chromosome 19p13.3, and no reports examined the role of lncRNA PLAC2 in glioma.

Ribosomal proteins (RPs) are essential components of the ribosome that comprise a family of RNA‐binding proteins involved in ribosome biogenesis and protein translation 13. RPs also have extra‐ribosomal functions independent of protein biosynthesis 14. For example, RPS15A is highly expressed in human glioblastoma; RPS15A knockdown inhibits human glioblastoma progression *in vitro* and *in vivo via* the AKT pathway 15. RPS11 and RPS20 overexpression at the transcriptional or translational level predicts decreased survival in patients with newly diagnosed primary glioblastoma 16. RPL36 may be involved in the early stages of hepatocarcinogenesis and can serve as an independent prognostic marker for resected hepatocellular carcinoma 17. Genome‐wide search for genes with aberrant methylation in colorectal cancer by a methylated DNA immunoprecipitation‐ChIP analysis found that RPL36 supposed to have critical roles in colorectal tumorigenesis 18. Studies reveal that RPL36 may restrain oncogenic Kras‐induced pancreatic tumour formation in zebrafish 19. However, the function and mechanism of action of RPL36 in glioma are unclear.

In this study, we report a novel lncRNA PLAC2 in glioma development and cell proliferation. PLAC2 expression was down‐regulated whereas that of RPL36 was up‐regulated in glioma as compared to normal brain tissues. PLAC2 overexpression inhibited tumour cell growth and induced cell cycle arrest in a (STAT)1/RPL36‐dependent manner *in vitro* and *in vivo*. These results provide new insight into glioma pathogenesis and a basis for the development of lncRNA‐based glioma therapies.

## Materials and methods

### Patients and specimens

Two independent cohorts of 61 glioma patients were enrolled in this study. In cohort 1, fresh glioma and adjacent non‐cancerous tissue specimens were collected from six patients who initially underwent surgery and were diagnosed with glioma at Nanfang Hospital, Southern Medical University (Guangzhou, China). In cohort 2, 55 glioma and 20 normal brain tissue specimens were obtained from patients who underwent surgery at the same hospital. None of the patients had received chemo‐ or radiotherapy, and pathological diagnoses were confirmed in all cases. Specimens were flash‐frozen at the time of collection and stored in liquid nitrogen. Written, informed consent was obtained from patients, and ethical consent was granted for the study by the Committees for Ethical Review of Research involving Human Subjects of Southern Medical University.

### Animals

Female BALB/C nude mice (5 weeks old; Vital river, Beijing, China) were maintained under specific pathogen‐free conditions. *In vivo* experiments were carried out according to our institution's guidelines for the use of laboratory animals and were approved by the Institutional Animal Care and Use Committee of Nanfang Hospital.

### Cell lines

U87MG and U251 glioma cell lines were obtained from the American Type Culture Collection (Manassas, VA, USA). Cells were cultured in Dulbecco's modified Eagle medium (DMEM; Gibco, Grand Island, NY, USA) supplemented with 10% foetal bovine serum (Gibco), 100 U/ml penicillin and 100 mg/ml streptomycin (Gibco) at 37°C in a humidified atmosphere of 5% CO_2_.

### Cell fractionation

Cells were grown in 15‐cm dishes (NUNC). Nuclear/cytoplasmic fractionation was performed using Cytoplasmic and Nuclear RNA Purification Kit (NORGEN, Thorold, Canada), following the manufacturer's protocol.

### RNA isolation and quantitative real‐time (qRT‐)PCR

Total RNA from cultured cells or human tissue was extracted with TRIzol reagent (Takara Bio, Otsu, Japan) according to the manufacturer's instructions. The cDNA for long non‐coding (lnc)RNA was synthesized using the All‐in‐One First‐Strand cDNA Synthesis kit (GeneCopoeia, Rockville, MD, USA) in a 20‐μl reaction containing 1000–2000 ng total RNA. The cDNA for mRNA was synthesized using the PrimeScript II 1st Strand cDNA Synthesis kit (Takara Bio) according to the manufacturer's instructions. Real‐time PCR was performed using a SYBR Green PCR kit (Takara Bio). Real‐time‐PCR was carried out on an ABI 7500 Fast Real‐Time PCR system (Applied Biosystems, Foster City, CA, USA). U6 and glyceraldehyde 3‐phosphate dehydrogenase (*GAPDH*) were used as internal controls for lncRNA and mRNA, respectively. Expression levels were quantitated with the ΔΔCt method. All samples were prepared in triplicate, and the mean value was used for comparative analyses. Primers are listed in Table S1.

### Western blot analysis

Cells were harvested and lysed in radioimmunoprecipitation assay buffer (KeyGen, Nanjing, China) containing protease and phosphatase inhibitors and phenylmethanesulfonyl fluoride (PMSF) (all from KeyGen). Protein concentration was determined using an enhanced Bicinchoninic Acid Protein Assay kit (KeyGen), and proteins were separated on a 12% sodium dodecyl sulphate polyacrylamide gel and transferred to a polyvinylidene difluoride membrane (Millipore, Billerica, MA, USA), which was blocked with 5% bovine serum albumin for 1 hr at room temperature and then incubated overnight at 4°C with primary antibodies. After incubation with appropriate horseradish peroxidase‐conjugated secondary antibodies, immunoreactivity was detected by enhanced chemiluminescence using the Western Blotting Substrate (Fdbio, Hangzhou, China). Protein expression level was quantified with Quantity One software (Bio‐Rad, Hercules, CA, USA) and normalized to the level of β‐actin. As for detection of STAT1 nuclear translocation, nuclear extracts were first prepared using a Nuclear and Cytoplasmic Protein Extraction Kit (Beyotime Biotechnology, Shanghai, China) and then subjected to Western blot analysis using a mouse antibody against STAT1, with lamin B as nuclear control.

### Cell proliferation assay

U87MG and U251 cells were seeded in 96‐well plates at a density of 2 × 10^3^ cells/well. A 10‐μl volume of Cell Counting Kit‐8 solution (Dojindo Laboratories, Kumamoto, Japan) was added to each well followed by incubation for 1.5 hrs at 37°C. Absorbance at 450 nm was measured on a Thermo Multiskan MK3 reader (Thermo Fisher Scientific, Waltham, MA, USA). Six replicate wells were prepared for each group, and five independent experiments were performed.

### Cell invasion assays

For the invasion assays, 5 × 10^4^ cells in 200 ml serum‐free medium were seeded into the upper chamber of a Transwell insert (8‐mm pore size; EMD Millipore, Billerica, MA, USA) coated with Matrigel. The lower chamber was filled with 600 litres medium containing 10% FBS. Following incubation in a humidified incubator at 37°C with 5% CO 2 for 12 hrs, the cells remaining on the upper membrane were removed with a cotton swab. The cells, which had migrated or invaded through the membrane, were stained with methanol and 0.1% crystal violet, images were captured, and the cells were counted using a BX51 microscope (Olympus, Tokyo, Japan). Invasion was assessed by counting the number of stained cell nuclei from 10 randomly selected fields per filter in each group (magnification, ×400), with the cell counts expressed as the mean number of cells per field of view. Three independent experiments were performed in triplicate.

### Flow cytometry

Cells (1 × 10^6^) were trypsinized and resuspended to obtain single‐cell suspensions. Detached cells were fixed overnight at 4°C in 70% ethanol, then stained with propidium iodide (Cell Cycle Detection kit; KeyGen) and analysed with a FACScan flow cytometer (BD Biosciences, San Jose, CA, USA) and ModFit 3.0 software (Verity Software House, Topsham, ME, USA). To detect apoptosis, cells were stained with fluorescein isothiocyanate‐conjugated Annexin V and propidium iodide (Apoptosis Detection kit; KeyGEN) as recommended by the manufacturer and analysed by flow cytometry. Data were analysed with FlowJo software (Tree Star, Ashland, OR, USA).

### Lentivirus (LV) construction and cell transfection

LV vectors (LV‐Mock and LV‐ PLAC2) were prepared as previously described 20. U87MG cells and U251 cells were cultured for 24 hrs before use in experiments. Cells grown to 50–70% confluence were transduced using polybrene reagent (Santa Cruz Biotechnology, Dallas, TX, USA) in DMEM according to the manufacturer's instructions at a multiplicity of infection of 1. Stable clones were selected after 2 weeks using geneticin (G418), and PLAC2 expression was evaluated by qRT‐PCR.

### Plasmid construction and short interfering (si)RNAs

Full‐length human ribosomal protein (RP)L36 cDNA was cloned into pFLAG‐CMV2 (Sigma–Aldrich, St. Louis, MO, USA) to generate the RPL36 vector. U87MG and U251 cells were seeded overnight and transfected the following day (at 50% confluence) with the RPL36 or control vector using Lipofectamine 2000 (Invitrogen, Carlsbad, CA, USA). siRNA sequences specific for signal transducer and activator of transcription (STAT)1 and RPL36 (Table S2) as well as scrambled siRNA were synthesized by Ribobio (Guangzhou, China) and were transfected into cells using Lipofectamine RNAiMAX Reagent (Invitrogen) according to the manufacturer's instructions. Transfected cells were grown for 48 hrs before analysis.

### Luciferase reporter assay

Cells seeded in 12‐well plates (3 × 10^5^ cells/well) were cotransfected with each of the indicated plasmids, a reporter plasmid (pGMSTAT1‐Lu) (Genomeditech, Shanghai, China) and an internal control plasmid (PGMR‐TK‐Renilla) (Genomeditech). After 24 hrs, the cells were harvested and assayed for luciferase activity. The luciferase assays were performed using a dual‐luciferase reporter assay system (Catalog#:E1910; Promega, Madison, WI, USA). Luciferase activity was measured by a Multifunctional microplate reader SpectraMax M5 (Molecular Devices, Sunnyvale, CA, USA). Firefly luciferase activity was normalized to Renilla luciferase activity for each sample.

### Immunohistochemistry

Frozen glioma or mouse tumour specimens were cut into 5‐μm sections and mounted on slides coated with poly‐l‐lysine (Sigma–Aldrich), air‐dried and fixed with acetone. Immunohistochemistry was performed as previously described 20, with minor modifications. Antibodies against the following proteins were used: RPL36 (ab209340; Abcam, Cambridge, UK), cyclin‐dependent kinase (CDK) 2 (BF0053; Affinity, Cincinnati, OH, USA) and Ki‐67 (ab15580; Abcam) (all at 1:100). Positive pixel intensity was measured as integrated optical density using Image‐Pro Plus v.6.2 software (Media Cybernetics, Rockville, MD, USA), with uniform settings applied to all slides. The area of positive immunoreactivity for each protein was calculated as the percentage of total section area. Values are expressed as mean ± standard deviation.

### Fluorescence *in situ* hybridization (FISH)

Paraffin‐embedded sections (4 μm thick) were deparaffinized, dehydrated, washed twice in distilled water for 2 min. and incubated in pre‐treatment solution (1 M NaSCN) at 80°C for 30 min. Sections were digested in 0.4 ml pepsin solution at 37°C for 15 min., rinsed twice in 2× saline sodium citrate (SSC) for 5 min., fixed in 4% formaldehyde for 10 min. at room temperature, dehydrated by immersion in 70%, 85% and 100% ethanol for 1min each at room temperature and then air‐dried. Probe mixture (aagcgggaattgcagggtagat with fam mark at the 5ꞌ, targeting PLAC2) was applied to each slide (10 μl/slide) followed by incubation in a humidified chamber at 73°C for 5 min. and at 37°C overnight for hybridization. The cover slips were removed, and slides were washed in 0.4× SSC for 2 min. at 72°C followed by 2× SSC for 2 min. at room temperature. Nuclei were counterstained with 4ꞌ,6ꞌ‐diamidino‐2‐phenylindole (DAPI; Invitrogen), and slides were stored in the dark at 4°C. FISH signals were visualized under a 710 NLO microscope (Olympus). A minimum of 100 tumour nuclei were evaluated for each specimen.

### Chromatin isolation by RNA purification (ChIRP)

ChIRP allows unbiased high‐throughput screening of RNA‐bound DNA and proteins 21. Briefly, cultured cells were cross‐linked *in vivo*, and chromatin was extracted and homogenized. Biotinylated complementary oligonucleotides tiling the RNA of interest were hybridized to target RNAs and isolated using magnetic streptavidin beads. Co‐purified chromatin was eluted for protein or DNA, which was used for identification and quantitation. Biotinylated 20 mer antisense oligonucleotides were designed using Stellaris single‐molecule FISH probe designer software (singlemoleculefish.com)^22^. ChIRP oligonucleotides are listed in Table S3 and a schematic illustration of 10 antisense DNA‐tiling probes binding to PLAC2 is shown in Fig. S3A. U87MG cells were cultured to confluence as previously described in tissue culture plates or flasks. After rinsing with PBS followed by trypsinization, resuspended cells were transferred to 50‐ml Falcon tubes and centrifuged at 800 g for 4 min. 20 million cells are typically sufficient for one ChIRP sample. The medium was removed, and cells (4 × 10^7^) were resuspended in 40 ml PBS. The cells were spun at 800 rcf for 4 min. The PBS was removed and cells were cross‐linked in 3% formaldehyde for 30 min., followed by quenching in 0.125 M glycine for 5 min. 23. Frozen cell pellets were thawed at room temperature and centrifuged at 2000 g for 3 min. at 4°C. The weight of each pellet was recorded, and a volume of lysis buffer 10 times greater than the mass of the pellet, containing protease inhibitor, PMSF and Superase‐in, was added followed by mixing; the pellet was then resuspended in 10 times the volume of lysis buffer followed by sonication (Bioruptor; Diagenode, Liège, Belgium) at 4°C for at least 30 min. (30 sec. on, 45 sec. off; output = 7) until the sample was clear. A 5‐μl volume of lysate was transferred to a fresh Eppendorf tube, and 90 μl proteinase K buffer and 5 μl proteinase K were added. After vortexing followed by centrifugation, the sample was incubated for 45 min. at 50°C. DNA was extracted using a PCR purification kit (Qiagen, Valencia, CA, USA) and visualized on a 1% agarose gel. Sonicated samples were centrifuged at 16,100 g for 10 min. at 4°C. Probes (100 pmol/ml of initial lysate before hybridization buffer) were added followed by incubation at 37°C for 4 hrs with shaking. The C‐1 magnetic beads (stored at 4°C) were prepared for hybridization; 100 μl per 100 pmol probes were added, followed by incubation at 37°C for 30 min. with shaking. The beads were washed five times with shaking for 5 min. at 37°C in pre‐warmed wash buffer consisting of 2× SSC, 0.5% sodium dodecyl sulphate (SDS), protease inhibitors, 1 mM PMSF and Superase‐in. After the final wash, the beads were resuspended in 1 ml wash buffer and separated for ChIP. qRT‐PCR detection of *RPL36* was carried out using the following two forward and reverse primer pairs: RPL36 BS, 5ʹ‐3ʹ TCTATGCTGGGGTTTGGGAAC and 5ʹ‐3ʹ CCACAGTTTGCGTTTGTGTG; and RPL36 B2, 5ʹ‐3ʹ AGAGCAAGACCCTGTCTGGA and 5ʹ‐3ʹ GTAGGGACGGGACGCTAAAT. Primers for GAPDH were 5ʹ‐3ʹ CGGCTACTAGCGGTTTTACG and 5ʹ‐3ʹ AAGAAGATGCGGCTGACTGT (Fig. S4B).

For protein elution, beads were collected and resuspended in biotin elution buffer composed of 12.5 mM biotin (Invitrogen), 7.5 mM HEPES (pH 7.5), 75 mM NaCl, 1.5 mM EDTA, 0.15% SDS, 0.075% sarkosyl and 0.02% Na‐deoxycholate and mixed at room temperature for 20 min. and 65°C for 10 min. The eluent was transferred to a fresh tube, and beads were again eluted. The two eluents were pooled, and residual beads were removed; trichloroacetic acid (25% of the total volume) was added, and proteins were precipitated overnight at 4°C. The following day, proteins were pelleted at 16,000 g and 4°C for 30 min.; the pellets were washed once with cold acetone and centrifuged again at 16,000 rcf and 4°C for 5 min. The acetone was removed, and the pellet was air‐dried for 1 min. at room temperature. Proteins were immediately solubilized in Laemmli sample buffer (Invitrogen) and boiled at 95°C for 30 min. with occasional mixing to reverse cross‐linking. Final protein samples were separated by Bis–Tris SDS polyacrylamide gel electrophoresis (Invitrogen) for mass spectrometry analysis.

### Chromatin immunoprecipitation (ChIP)‐PCR

ChIP‐PCR was performed as previously described 20. Briefly, cells were seeded on coverslips and washed with PBS. ChIP was carried out using an anti‐STAT1 antibody (AHO0832; Thermo Fisher Scientific) and the Pierce Agarose ChIP kit (#26156; Thermo Fisher Scientific) according to a published protocol 24. DNA was analysed on a LightCycler 480 (Roche Diagnostics, Indianapolis, IN, USA) using LightCycler 480 SYBR Green I Master Mix (#4707516001; Roche Diagnostics) according to the manufacturer's instructions. qRT‐PCR detection of *RPL36* and *GAPDH* was performed using the same primers as those used in ChIRP‐PCR.

### RNA immunoprecipitation (RIP)‐PCR

For RNA immunoprecipitation, nuclear extracts were prepared as previously described 25. Briefly, 100 μl of nuclear extract was diluted to 1 ml with nuclear lysis buffer; samples were incubated overnight at 4°C with anti‐STAT1 antibody (AHO0832; Thermo Fisher Scientific) or non‐specific mouse IgG. Protein G Plus Protein A agarose beads (Calbiochem, San Diego, CA, USA) were added for 1–2 hrs at 4°C. After washing and elution, immunoprecipitated RNA was extracted with TRIzol reagent (Takara Bio) and dissolved in nuclease‐free water. qRT‐PCR was performed to measure the levels of PLAC2 associated with STAT1. PCR products were separated on a 2% agarose gel. Primer sequences are shown in Table S4.

### *In vivo* tumourigenicity assay

Female BALB/C nude mice were subcutaneously injected in the underarm area with a suspension of 1 × 10^7^ cells in 200 μl of PBS. The mice were observed 4 weeks before killing and recovery of the tumours. The wet weight of each tumour was determined and a portion was fixed in 4% paraformaldehyde and embedded in paraffin for haematoxylin and eosin staining. Experiments were performed according to institutional guidelines for the use of laboratory animals and were approved by the institutional Animal Care and Use Committee of Nanfang Hospital.

### Statistical analysis

Statistical analyses were performed using SPSS v.20.0 (Abbott Laboratories, Chicago, IL, USA) and GraphPad v.6.0 (GraphPad Inc., La Jolla, CA, USA) software. Data are expressed as the mean ± SEM of at least three independent experiments for each group. The χ^2^ test was used to examine the relationship between PLAC2 expression level and clinicopathological characteristics. A two‐tailed Student's *t*‐test was used to compare two independent groups. One‐way analysis of variance was used to assess differences between groups for all *in vitro* analyses. The correlation between PLAC2 and RPL36 expression was analysed by Spearman rank correlation 26. A *P* value <0.05 was considered significant.

## Results

### LncRNA PLAC2 is strongly down‐regulated in glioma tissues and inhibits glioma cell proliferation, induces cell cycle arrest

**Figure 1:**
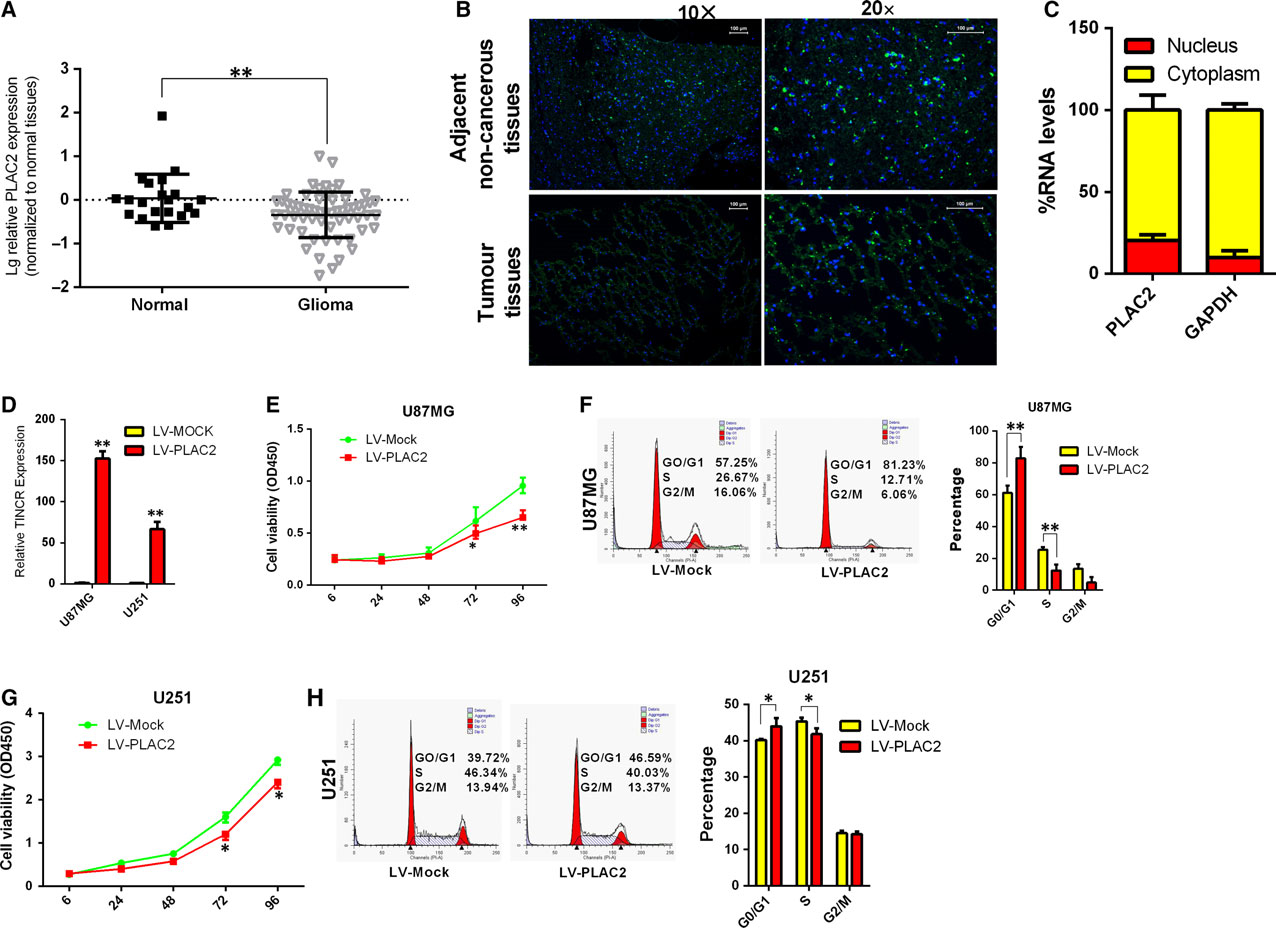
LncRNA PLAC2 is strongly down‐regulated in glioma tissues and inhibits glioma cell proliferation, induces cell cycle arrest. (**A**) PLAC2 was down‐regulated in glioma as compared to normal brain tissues. Log10 transformation was applied to expression levels. *P < 0.05, **P < 0.01. (**B**) Representative images of PLAC2 expression in glioma (Tumour) and adjacent non‐cancerous tissue by FISH (*n* = 6). (**C**) Percentage of nuclear and cytoplasmic RNA levels of PLAC2 and GAPDH measured by qRT‐PCR after cell fractionation in U87MG cells. The graph shows the average of three independent experiments. (**D**) Stable overexpression of PLAC2 in U87MG and U251 cells after lentiviral infection with LV‐PLAC2. Control cells were infected with the empty lentiviral vector LV‐Mock. Experiments were performed in triplicate. **P < 0.01. (**E**–**H**) U87MG and U251 cells were treated with LV‐Mock or LV‐PLAC2 and cell viability and cell cycle phase were evaluated with the CCK‐8 assay (**E** and **G**) and by FACS (**F** and **H**), respectively. PLAC2 overexpression in U87MG and U251 cells decreased cell proliferation and increased G1/S arrest relative to control cells. *P < 0.05, **P < 0.01.

Our lncRNA expression determined using the Arraystar Human LncRNA Array v2.0 platform in three paired tumour and non‐tumour glioma tissues revealed that lncRNA PLAC2 expression was down‐regulated in glioma tissues (fold change = 2.694; *P* < 0.001). The expression of PLAC2 was confirmed by quantitative real‐time (qRT‐)PCR in 20 additional normal brain and 55 glioma tissue samples. PLAC2 expression was strongly down‐regulated (Fig. 1A; *P* < 0.01) in glioma tissues; this was confirmed by fluorescence *in situ* hybridization analysis of paraffin‐embedded glioma and paired normal specimens (Fig. 1B; *P* < 0.05). Further characterization of PLAC2 revealed that it is located in Chromosome 19 – NC_000019.10 (5558167..5568034) (Fig. S1A) and located both in the cytoplasm and the nucleus (Fig. 1C). To investigate the role of PLAC2 in glioma, we evaluated the association between PLAC2 level and clinicopathologic characteristics of glioma patients (Table 1). A negative correlation was observed between expression of PLAC2 and the proliferation marker Ki‐67 (*P* < 0.05), suggesting that PLAC2 plays a critical role in glioma development. Stable cell line overexpression of PLAC2 in U87MG and U251 cells after lentiviral infection with LV‐PLAC2 was established (Fig. 1D). Lentivirus‐mediated PLAC2 overexpression suppressed proliferation in both cell types, as determined with the Cell Counting Kit‐8 assay (Fig. 1E and G). The number of colonies in PLAC2‐overexpressing cells was lower than in the control group (Fig. S1B). A flow cytometry analysis of cell cycle distribution revealed that the percentage of cells in S and G1 phases was decreased and increased, respectively, upon overexpression of PLAC2 in both cell lines (Fig. 1F and H), whereas no differences were observed in terms of cell invasiveness and rate of apoptosis (Fig. S1C and D).

**Table 1:** Clinical and molecular pathology features of Glioma samples in association with PLAC2 expression

	Low	High	*P*
Gender, female/male	12/19	8/9	0.575
Age at diagnosis, y	40.6 ± 19.3	36.6 ± 20.9	0.514
IDH1 mutation (no mutation/mutation)	8/6	6/5	0.897
MGMT promoter methylation (unmethylation/methylation)	8/5	6/9	0.256
Ki‐67 (low/high)	6/18	10/5	0.010^a^

a *P* values less than 0.05 were considered statistically significant.

### PLAC2 overexpression inhibits cell proliferation and induces G1/S arrest *via* modulation of RPL36 expression

**Figure 2:**
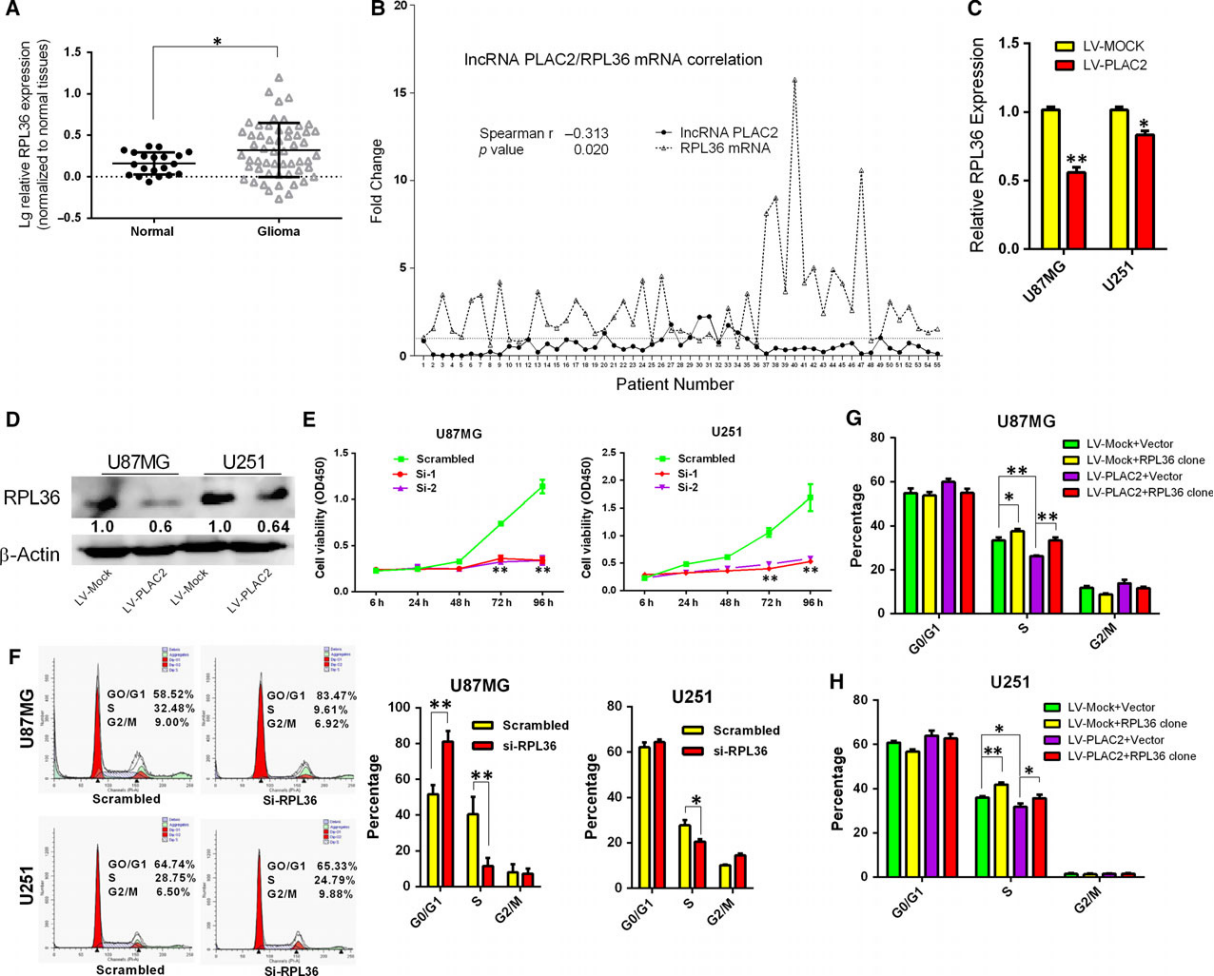
PLAC2 overexpression inhibits cell proliferation and induces G1/S arrest *via* modulation of RPL36 expression. (**A**) RPL36 mRNA expression was up‐regulated in glioma as compared to normal brain tissues. *P < 0.05. (**B**) Fold change and correlation of PLAC2 and RPL36 mRNA levels in 55 glioma specimens by Spearman's correlation test. Relative fold change in expression was calculated with the 2^−ΔΔCt^ method. *P < 0.05. (**C**,** D**) RPL36 mRNA (**C**) and protein (**D**) levels were markedly reduced by lentivirus‐mediated PLAC2 overexpression. Experiments were performed in triplicate (*n* = 3). *P < 0.05, **P < 0.01. (**E**–**F**) U87MG and U251 cells were treated with siRNA against RPL36, and cell viability and cell cycling were measured with the CCK‐8 assay (**E**) and by FACS (**F**), respectively. RPL36 knockdown inhibited proliferation and induced G1/S cell cycle arrest relative to control cells. Si‐1 and Si‐2 were the oligo used to knock down RPL36, Si‐RPL36 represents Si‐1; scrambled was the negative control. *P < 0.05, **P < 0.01. (**G**–**H**) Changes in the cell cycle after cotransfection of U87MG and U251 cells with indicated treatment. RPL36 overexpression in PLAC2‐overexpressing cells rescued G1/S arrest. *P < 0.05, **P < 0.01.

Recent studies indicate that lncRNA function can be inferred from its location and will probably affect expression of nearby protein‐coding genes 27. Next, we performed Pearson correlation analysis between differentially expressed mRNA transcripts near to the differentially expressed lncRNAs (Pearson correlation, >0.9 or <−0.9; distance, <100 kb). Thus, we hypothesized that PLAC2 might negatively regulate RPL36 expression in cis. Next, we analysed RPL36 mRNA levels by qRT‐PCR in the same 20 additional normal brain and 55 glioma tissue samples and found that RPL36 was up‐regulated in glioma tissues (Fig. 2A). Pearson correlation analysis revealed that there is a negative correlation between *PLAC2* and *RPL36* mRNA levels in glioma tissues (Spearman *r* = −0.313, *P* < 0.05; Fig. 2B). Moreover, Western blot analysis and immunohistochemistry revealed that the protein level of RPL36 in glioma tissues were up‐regulated (Fig. S2A and B). Patients were divided into RPL36 high and low expression groups based on mRNA levels, and the association between *RPL36* transcript expression in glioma tissues and clinicopathological characteristics was assessed. High *RPL36* mRNA level was positively associated with Ki‐67 expression (*P* < 0.05, Table S1). In addition, RPL36 mRNA and protein levels were markedly reduced by lentivirus‐mediated PLAC2 overexpression (Fig. 2C and D; *P* < 0.05). These results indicate that PLAC2 negatively regulates RPL36 expression.

To investigate the biological significance of RPL36 expression in glioma development and progression, *RPL36* expression was suppressed by short interfering (si)RNA‐mediated knockdown (Table S3 and Fig. S2C). Proliferation was inhibited and the percentage of cells in S phase was decreased in U87MG and U251 cells (Fig. 2E and F). Flow cytometry analysis revealed that *RPL36* knockdown increased apoptosis relative to the control (Fig. S2E) but had no effect on migration (Fig. S2D). RPL36 overexpression by transfection of a plasmid expressing RPL36 induced cell cycle progression in both cell lines (Fig. S2F and G).

To determine whether the cell cycle arrest induced by PLAC2 in U87MG and U251 cells is RPL36‐dependent, cells were infected with PLAC2 lentivirus along with a vector for overexpression or knockdown of RPL36. SiRNA‐mediated knockdown of RPL36 combined with PLAC2 overexpression increased the percentage of cells arrested at G1/S phase (Fig. S2H), an effect that was partially rescued by RPL36 overexpression (Fig. 2G and H). These findings suggest that PLAC2 inhibits cell proliferation and induces S‐phase arrest *via* modulation of RPL36 expression.

To investigate the mechanism by which PLAC2 regulates cell cycling, we evaluated the expression of genes involved in the cell cycle by qRT‐PCR 28. The expression of CDK4, cyclin D1, and most prominently CDK2 was reduced in PLAC2 lentivirus‐treated U87MG cells (Fig. S3A). CDK2 regulates the G1/S transition 28. The protein level of CDK2 was decreased by PLAC2 overexpression in U87MG and U251 cells (Fig. S3B, *P* < 0.05). Transfection of siRNA targeting RPL36 reduced CDK2 expression, while the opposite effect was observed using a plasmid encoding RPL36 (Fig. S3C; *P* < 0.05). *RPL36* knockdown in cells overexpressing PLAC2 cells further decreased the CDK2 level (Fig. S3D; *P* < 0.05). These findings suggest that CDK2 induces G1/S arrest as a result of PLAC2‐mediated suppression of RPL36.

### PLAC2 inhibits tumour growth *in vivo*

**Figure 3:**
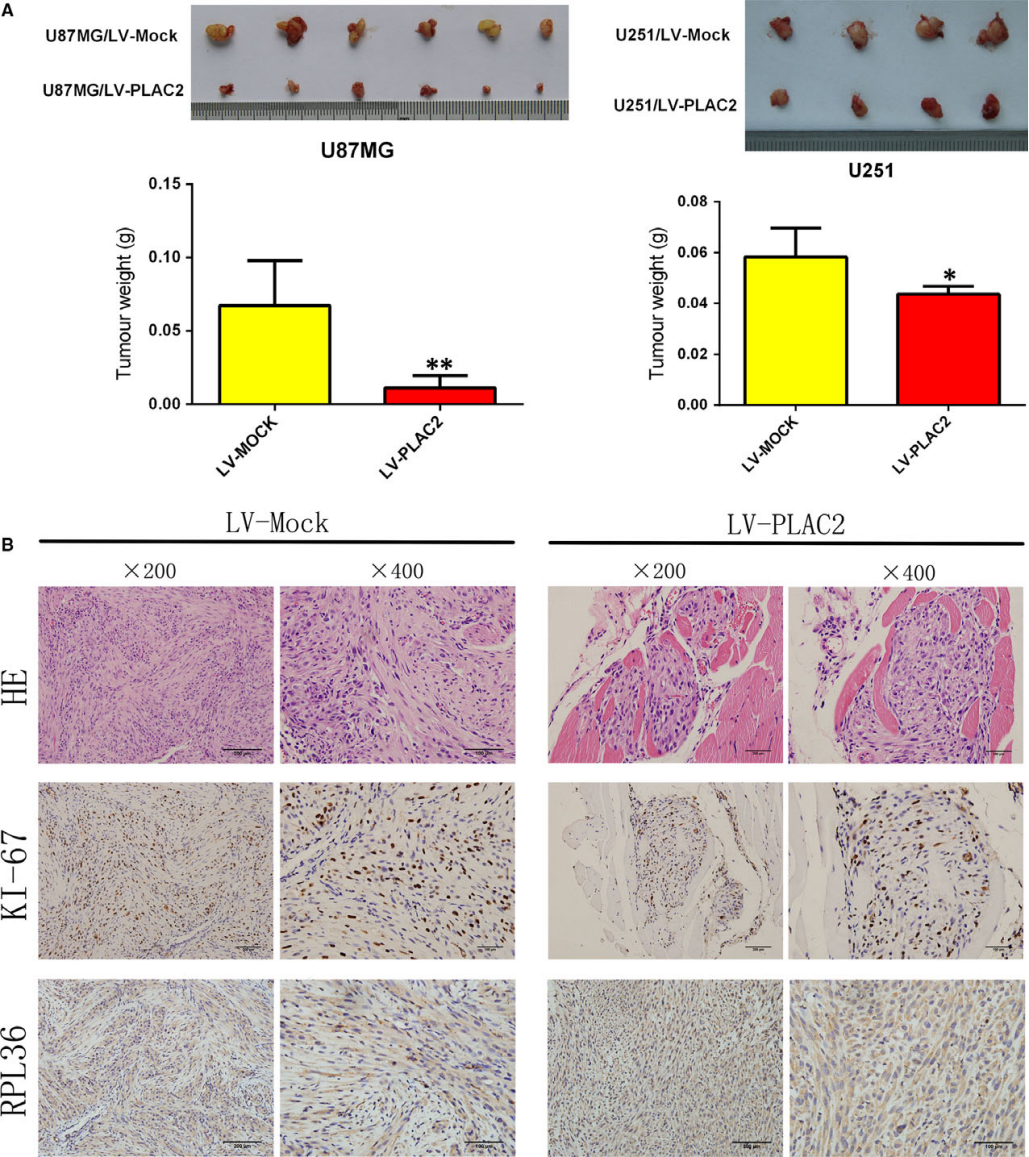
PLAC2 overexpression suppresses tumour growth *in vivo*. (**A**) Above: Representative images of tumours formed in nude mice injected subcutaneously with U87MG and U251 cells overexpressing PLAC2. Below: Tumour weights. *P < 0.05, **P < 0.01. (**B**) Representative images (200× and 400×) of haematoxylin‐stained section with immunohistochemical detection of Ki‐67, and RPL36 in paraffin‐embedded sections obtained from xenografts. The expression of all two factors was down‐regulated by PLAC2 overexpression relative to the control (*n* = 5).

To confirm the antitumorigenic effects of PLAC2 *in vivo*, we established a mouse xenograft model by subcutaneous injection of U87MG and U251 cells stably overexpressing PLAC2 into the right axilla; cells treated with a mock vector were injected into the left axilla as a control. The growth and weight of tumours overexpressing PLAC2 were lower than in control tumours (Fig. 3A). Moreover, PLAC2‐overexpressing tumours showed reduced expression of Ki‐67 and RPL36 as detected by immunohistochemistry (Fig. 5B; *P* < 0.05). These results indicate that PLAC2 inhibits tumour growth *in vivo*.

### PLAC2/STAT1 interaction regulates RPL36 expression

**Figure 4:**
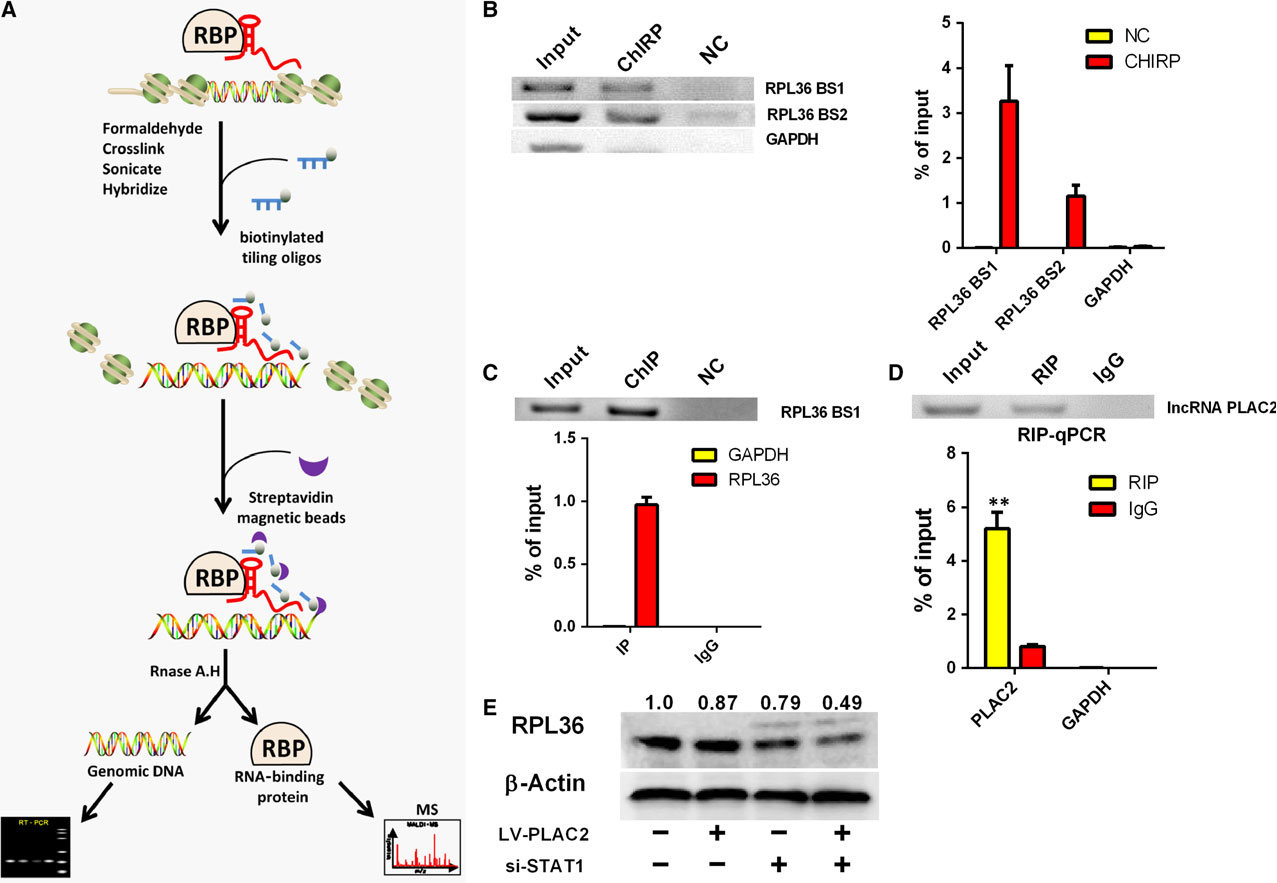
PLAC2 interacts with the transcription factor STAT1. (**A**) Workflow of ChIRP. Chromatin was cross‐linked to lncRNA:protein adducts *in vivo*. Biotinylated tiling probes were hybridized to the target lncRNA, and chromatin complexes were purified using magnetic streptavidin beads, followed by stringent washes. (**B**) Left: Detection of RPL36 DNA in the extracted DNA fraction of ChIRP samples by northern blotting; GAPDH DNA was not detected in the extract; Right: qRT‐PCR analysis revealed an enrichment of RPL36 DNA in the extracted DNA fraction of ChIRP samples. RPL36 DNA was amplified using two promoter‐specific forward and reverse primer pairs, RPL36 BS1 and RPL36 BS2. (**C**) U87MG cell lysates were subjected to ChIP using an anti‐STAT1 antibody. Top: Northern blot analysis of RPL36 DNA expression. Below: qRT‐PCR analysis of RPL36 DNA expression; >97% of RPL36 DNA was pulled down from the cell by hIP, while no GAPDH was detected. (**D**) Top: Detection of PLAC2 in the extracted RNA fraction of RIP samples, as detected by Northern blotting. Below: qRT‐PCR analysis of PLAC2 in the extracted RNA fraction of RIP samples, **P < 0.01. (**E**) STAT1 knockdown in PLAC2‐overexpressing cells enhanced the decrease in RPL36 level in U87MG cells. β‐Actin was used as a loading control.

LncRNAs mainly regulate their targets by affecting gene transcription 29. To examine the mechanism by which PLAC2 regulates RPL36 expression, we carried out chromatin isolation by RNA precipitation–mass spectrometry (ChIRP‐MS) using U87MG cell extracts (Fig. 4A, Fig. S4A–C). Bands corresponding to PLAC2‐bound proteins were evaluated by MS. Biotin‐labelled nonsense RNA with a length similar to PLAC2 was used as a negative control. The functions of proteins identified by ChIRP‐MS were determined by Gene Ontology analysis (Fig. S4D). Given that PLAC2 was found to regulate the mRNA levels of RPL36, we focused our search on known transcription factors. STAT1 had high frequency and score, and a bioinformatic analysis 30 confirmed that the *RPL36* transcript contains a STAT1‐binding site (Fig. S5C). Our microarray data revealed that STAT1 was up‐regulated in glioma tissues (fold change = 2.193; *P* < 0.05), whereas a tandem mass spectrum analysis also identified STAT1 as a potential binding partner of RPL36 (Fig. S5A). The DNA fraction from ChIRP samples were quantitated by PCR, revealing a significant enrichment of *RPL36* DNA (Fig. 4B).

**Figure 5:**
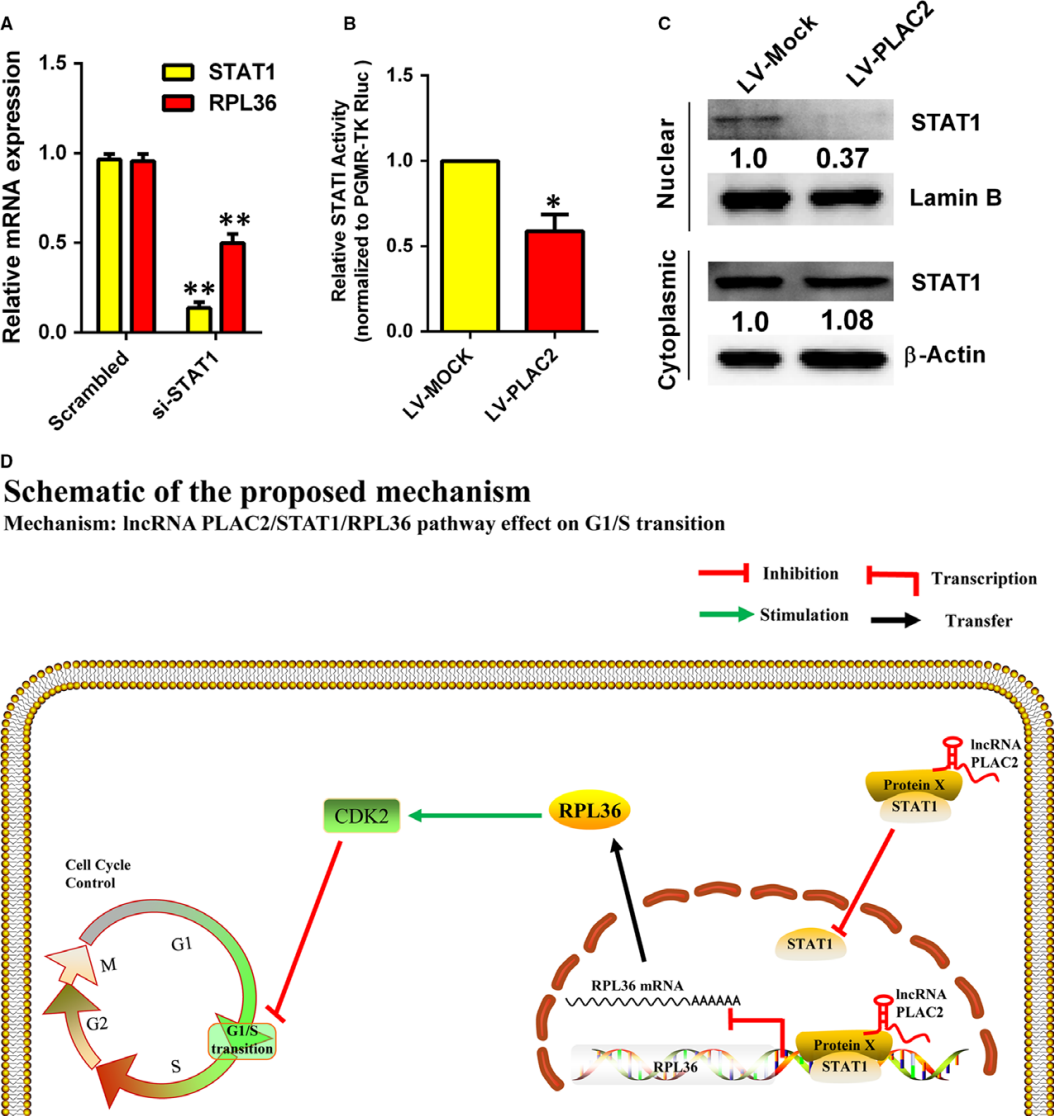
A schematic model of lncRNA PLAC2 functions during the cell cycle arrest. (**A**) RPL36 mRNA level was down‐regulated in U87MG cells upon STAT1 knockdown. Experiments were performed in triplicate (*n* = 3). **P < 0.01. (**B**) STAT1 transcription activity was reduced by lentivirus‐mediated PLAC2 overexpression. Luciferase activity was measured and normalized to Renilla luciferase. Data represent means SEM of data of triplicate cultures from three independent experiments, *P < 0.05. (**C**) Nuclear and cytoplasmic STAT1 protein were isolated and determined by Western blot. Expressions of nuclear STAT1 were decreased by lentivirus‐mediated PLAC2 overexpression while cytosolic STAT1 were not changed. Lamin B was used as normalized control in nuclear protein. β‐Actin was chosen as normalized control in cytoplasmic protein. Experiments were performed in triplicate (*n* = 3), *P* < 0.05. (**D**) Schematic illustration of the regulation of glioma progression by PLAC2/RPL36/STAT1/CDK2. The nucleus lncRNA PLAC2 bind with STAT1 and interact with RPL36 promoters, but the cytoplasmic lncRNA PLAC2 inhibited STAT1 nuclear transfer, thereby decreasing RPL36 expression, inhibiting cell proliferation, and inducing cell cycle arrest. Protein X is an as yet unidentified protein involved in this regulatory network.

To confirm the interaction between RPL36 and STAT1, we carried out chromatin immunoprecipitation‐ and RNA immunoprecipitation (RIP)‐PCR assays using an anti‐STAT1 antibody. STAT1 protein was found to interact with RPL36 promoter (Fig. 4C), while RIP‐PCR revealed that PLAC2 was present in the RNA fraction of RIP samples (Fig. 4D). To determine whether PLAC2 associates with STAT1 to modulate RPL36 expression, RPL36 protein levels were measured upon STAT1 knockdown. Loss of STAT1 in cells overexpressing PLAC2 further decreased RPL36 level in U87MG cells (Fig. 4E), but there is no feedback adjustment between RPL36 and STAT1 (Fig. S5D). In addition, the RPL36 mRNA level was decreased by silencing STAT1 (Fig. 5A), which suggest that STAT1 is a positive regulator of RPL36 transcription. Meanwhile, the STAT1 transcriptional activity was decreased by PLAC2 overexpression (Fig. 5B). To investigate whether decreased STAT1 transcriptional activity due to the level of STAT1 in cell nucleus, the distribution of STAT1 in cytoplasm and nucleus was detected. Our results revealed that STAT1 nuclear localization was drastically decreased by PLAC2 overexpression (Fig. 5C). Collectively, these data demonstrate that the nuclear PLAC2 bind with STAT1 and interact with RPL36 promoters, but the cytoplasmic PLAC2 inhibited STAT1 nuclear transfer, thereby decreasing RP36 expression, inhibiting cell proliferation and inducing cell cycle arrest. Schematic illustration of the regulation of glioma progression by PLAC2/RPL36/STAT1/CDK2 is showed in Fig. 5D.

## Discussion

Gliomas are the most common type of primary intracranial tumour 31. Despite the availability of treatments such as surgery and chemoradiotherapy, glioma incidence and mortality rates remain high 32. Clarifying the pathogenesis of this malignancy can lead to the development of more effective treatments. In the present study, we found that overexpression of PLAC2 blocked tumour cell proliferation and cell cycle progression *in vitro* and tumorigenesis *in vivo* by decreasing the level of RPL36 *via* interaction with STAT1. RPL36 knockdown had similar effects, which were exerted by suppression of CDK2 expression.

LncRNAs are active biological molecules 33, 34 that play important regulatory roles in many tumours 35, 36, 37. Recent studies have shown that lncRNAs regulate a wide variety of biological processes in glioma 26, 38, 39. We found here that PLAC2 expression was down‐regulated in glioma as compared to normal brain tissue. Moreover, these patients had higher expression of Ki‐67 than subjects with high PLAC2 levels. PLAC2 overexpression inhibited tumour cell proliferation and led to G1/S arrest. In addition, xenograft tumours grown from cells overexpressing PLAC2 had smaller mean volumes and weights than those grown from untransfected control cells. It has been shown that misexpression of lncRNAs contributes to numerous diseases. In addition, further study of lncRNA motifs could yield new RNA‐based targets for the prevention and treatment of human disease 12. In conclusion, lncRNA PLAC2 play an important role in glioma proliferation and can serve as a potential target for glioma therapy.

LncRNAs inhibit the expression of neighbouring protein‐coding genes in cis or in trans 20, 40, 41, 42. A bioinformatic analysis revealed that *RPL36* was located near *PLAC2* and was transcribed in the opposite direction to *PLAC2*. We observed a correlation between PLAC2 and *RPL36* mRNA expression in clinical glioma specimens. In addition, *RPL36* transcription was inhibited by PLAC2 overexpression. Previous studies have reported that cell cycle progression is blocked by loss of RPS6 43, 44. In our study, *RPL36* knockdown inhibited cell proliferation and enhanced PLAC2 overexpression‐induced cell cycle arrest; this effect was partly abolished by RPL36 overexpression. These results indicate that the effects of PLAC2 in glioma are RPL36‐dependent, which provide a new function pathway for cell cycle in glioma. In addition to the function of those RPs in ribosome assembly and protein translation, their ribosome‐independent functions have been little explored 43. In this study, we have provided evidence that the RPL36 is not just a housekeeping factor but is actively involved in the regulation of cell proliferation both *in vitro* and *in vivo*. However, there are conflicting data concerning its role in cancers. There are researches found that RPL36 may be involved in the progression of hepatocarcinogenesis and colon cancer 17, 45. However, Elayne Provost showed that rpl36, but not rpl23, effectively restrains oncogenic Kras‐induced pancreatic tumour formation in zebrafish 19. It may be tempting to reason that RPL36 may play different role in diverse cancers due to different forms of genetic and epigenetic restraint. These studies indicate that the functions of RPL36 in human cancers need to be further elucidated and stimulate study of the role of ribosomal proteins in multi‐factorial mechanisms of cancer. RPL36 has the potential to be applied to the development of ribosomal stress‐based cancer therapeutics.

CDK2 is a serine/threonine protein kinase that plays a key role in the G1/S transition 46. In the present study, CDK2 level was reduced by PLAC2 overexpression, an effect that was enhanced by *RPL36* knockdown. These results indicate that G1/S arrest and CDK2 down‐regulation induced by PLAC2 overexpression is mediated by RPL36. RPS6 activity is under cell cycle control 47. RPL31 has been shown to suppress cell growth and cycling *via* p53 signalling 48; induction of p21 by p53 following DNA damage inhibited CDK2 activity 49. Determining whether cell cycle regulation by RPL36 is mediated by inhibition of CDK2 by p53 and p21 is a question that will be the focus of future studies.

LncRNAs regulate the expression of target genes through various mechanisms 50, 51, including transcriptional regulation *via* physical interaction with the target gene promoter. In the present study, we investigated whether PLAC2 regulates RPL36 transcription by ChIRP‐MS analysis. Among the transcription factors identified by MS, STAT1 had high frequency and score; moreover, STAT1 was found to bind the *RPL36* promoter. PLAC2 and STAT1 were also found to interact by RIP‐PCR. STAT1 knockdown enhanced the inhibitory effect of PLAC2 overexpression on RPL36 level. These data demonstrate that the nuclear PLAC2 bind with STAT1 and interact with RPL36 promoters. Meanwhile, cytoplasmic PLAC2 inhibited nuclear translocation of STAT1. Thus, interaction between PLAC2 and STAT1 modulates RPL36 expression, suggested a new mechanism of how lncRNAs regulate the expression of nearby protein‐coding genes. STATs are usually activated by phosphorylation events and dimerization. How do the results here correlate with canonical Jak/STAT signalling needs further research. Elucidating the nature of the interaction between PLAC2 and STAT1 can provide a basis for new drugs for glioma treatment.

In summary, our results demonstrate that PLAC2 acts as a tumour suppressor that is down‐regulated in glioma. PLAC2 suppressed tumour cell proliferation in an RPL36‐dependent manner and induced cell cycle arrest *via* down‐regulation of CDK2. The cytoplasmic lncRNA PLAC2 inhibited STAT1 nuclear transfer, and interaction between nuclear PLAC2 and STAT1 modulated RPL36 transcription. These results indicate that PLAC2, RPL36, STAT1 and CDK2 form a regulatory network that controls cell cycle progression in glioma that can serve as a potential target for glioma therapy.

## Conflict of interest

The authors declare no competing financial interests.

## Supporting information


**Fig. S1** (**A**) PLAC2 and RPL36 genomic loci on chromosome 19. RPL36 is located near PLAC2 and is transcribed in the opposite direction. (**B**) PLAC2 overexpression decreased colony numbers in U251 cells relative to the control. (**C**) Cells overexpressing PLAC2 were stained with a combination of Annexin V and 7‐aminoactinomycin D and analyzed by FACS. Apoptosis rates did not differ significantly between the two groups in either U87MG or U251 cells. (**D**) PLAC2 overexpression had no effect on U87MG cell migration and invasion. LV‐PLAC2 and LV‐Mock are LV expressing PLAC2 and the empty lentivirus vector used as a control, respectively.

**Fig. S2** (**A**) Western blot analysis of RPL36 in glioma (T1–T6) and normal brain tissue (*n* = 6). Experiments were performed in triplicate. (**B**) Representative images of RPL36 expression in glioma (Tumor) and adjacent non‐cancerous tissue by immunohistochemistry (*n* = 6). (**C**) RPL36 mRNA and protein levels were downregulated in U87MG and U251 cells upon RPL36 knockdown by si‐1 or ‐2. Scrambled siRNA served as a negative control. (**D**) There was no difference in the migration of U87MG and U251 cells transfected with si‐RPL36 as compared to the respective controls, as determined by the transwell assay. (**E**) RPL36 knockdown increased the rate of apoptosis of U87MG and U251 cells. (**F**) RPL36 protein level was upregulated in U87MG and U251 cells upon transfection of plasmid expressing RPL36. *P < 0.05, **P < 0.01. (**G**) RPL36 overexpression induced cell cycle progression in both cell lines relative to control cells. RPL36 clone was the RPL36 expression plasmid and vector was the empty plasmid used as a control. *P < 0.05. (**H**) Changes in the cell cycle after cotransfection of U87MG and U251 cells with indicated treatment. RPL36 knockdown in PLAC2‐overexpressing cells enhanced G1/S arrest.

**Fig. S3** PLAC2/RPL36 pathway induces G1/S arrest *via* suppression of CDK2. (**A**) Relative expression levels of cell cycle‐associated genes were detected in U87MG cells overexpressing PLAC2 by qRTPCR. *P < 0.05, **P < 0.01. (**B**) U87MG and U251 cells were infected with LV expressing PLAC2 or an empty LV vector as a control, and CDK2 expression was evaluated by western blotting. The level was downregulated by PLAC2 overexpression. (**C**) CDK2 levels detected by western blotting after RPL36 knockdown or overexpression. CDK2 was upregulated in cells overexpressing RPL36 and was downregulated upon RPL36 knockdown. (**D**) CDK2 protein level was decreased by PLAC2 overexpression, an effect that was enhanced by RPL36 knockdown.

**Fig. S4** Schematic illustration of probes used for pulldown of PLAC2 and results of ChIRPMS. (**A**) Schematic illustration of the binding sites of 10 antisense DNA tiling probes on PLAC2. (**B**) MS data analysis performed using Matrix Science (http://www.matrixscience.com/). The number of hits represent the number of proteins obtained by ChIRP. (**C**) Proteins pulled down by ChIRP with a non‐targeting probe used as a control (NC), as visualized by silver staining. (**D**) Molecular functions or proteins identified by ChIRP‐MS according to Gene Ontology analysis.

**Fig. S5** (**A**) Mass spectra of STAT1. (**B**) PCR primers designed with the transcription start site at −2000 to +200 bp as template amplifying a product of 100–200 bp. Schematic illustrations of primers amplifying RPL36 (above) and GAPDH (below) are shown. (**C**) Schematic illustration of putative STAT1 binding sites in RPL36. (**D**) Transfection of siRNA targeting RPL36 in U87MG cells does not have a significant effect on STAT1 expression.

**Table S1** Clinical and molecular pathology features of Glioma samples in association with RPL36 mRNA expression
**Table S2** Sequences of primers used in this study
**Table S3.** Sequences of siRNAs used in this study
**Table S4** Antisense DNA tiling probes for pulldown of *PLAC2* or nonsense DNA tiling probes used for ChIRP
**Table S5** Sequences of primers used for RIP‐PCR
