# Supplementary figures and images for: LncRNA PLAC2 down‐regulates RPL36 expression and blocks cell cycle progression in glioma through a mechanism involving STAT1
Source: J Cell Mol Med. 2017 Sep 18;22(1):497–510. doi: 10.1111/jcmm.13338 (PMC5742712; doi:10.1111/jcmm.13338)

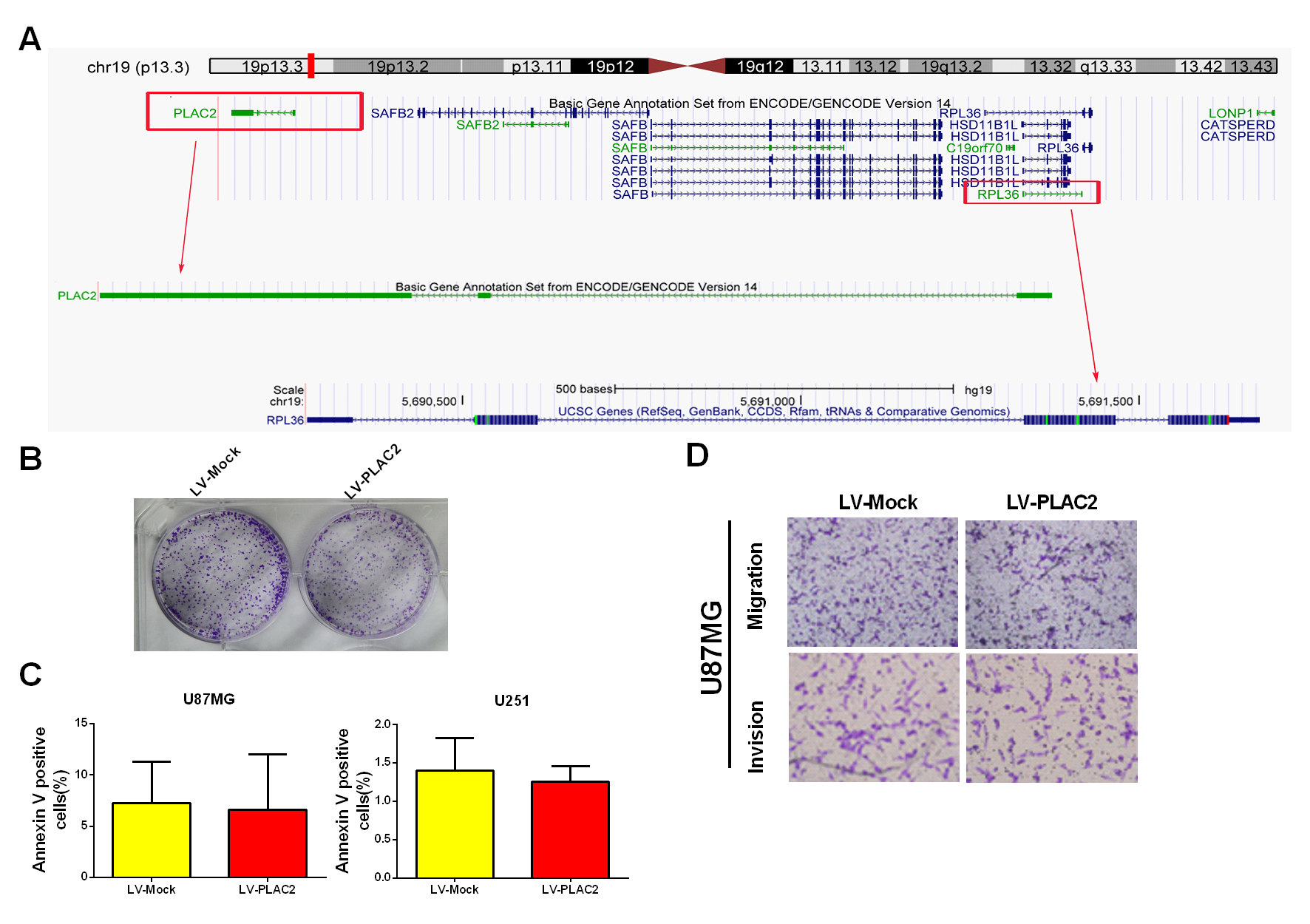

Supplement: Supplementary file 1 — Fig. S1 (A) PLAC2 and RPL36 genomic loci on chromosome 19. RPL36 is located near PLAC2 and is transcribed in the opposite direction. (B) PLAC2 overexpression decreased colony numbers in U251 cells relative to the control. (C) Cells overexpressing PLAC2 were stained with a combination of Annexin V and 7‐aminoactinomycin D and analyzed by FACS. Apoptosis rates did not differ significantly between the two groups in either U87MG or U251 cells. (D) PLAC2 overexpression had no effect on U87MG cell migration and invasion. LV‐PLAC2 and LV‐Mock are LV expressing PLAC2 and the empty lentivirus vector used as a control, respectively. [file JCMM-22-497-s001.tif]

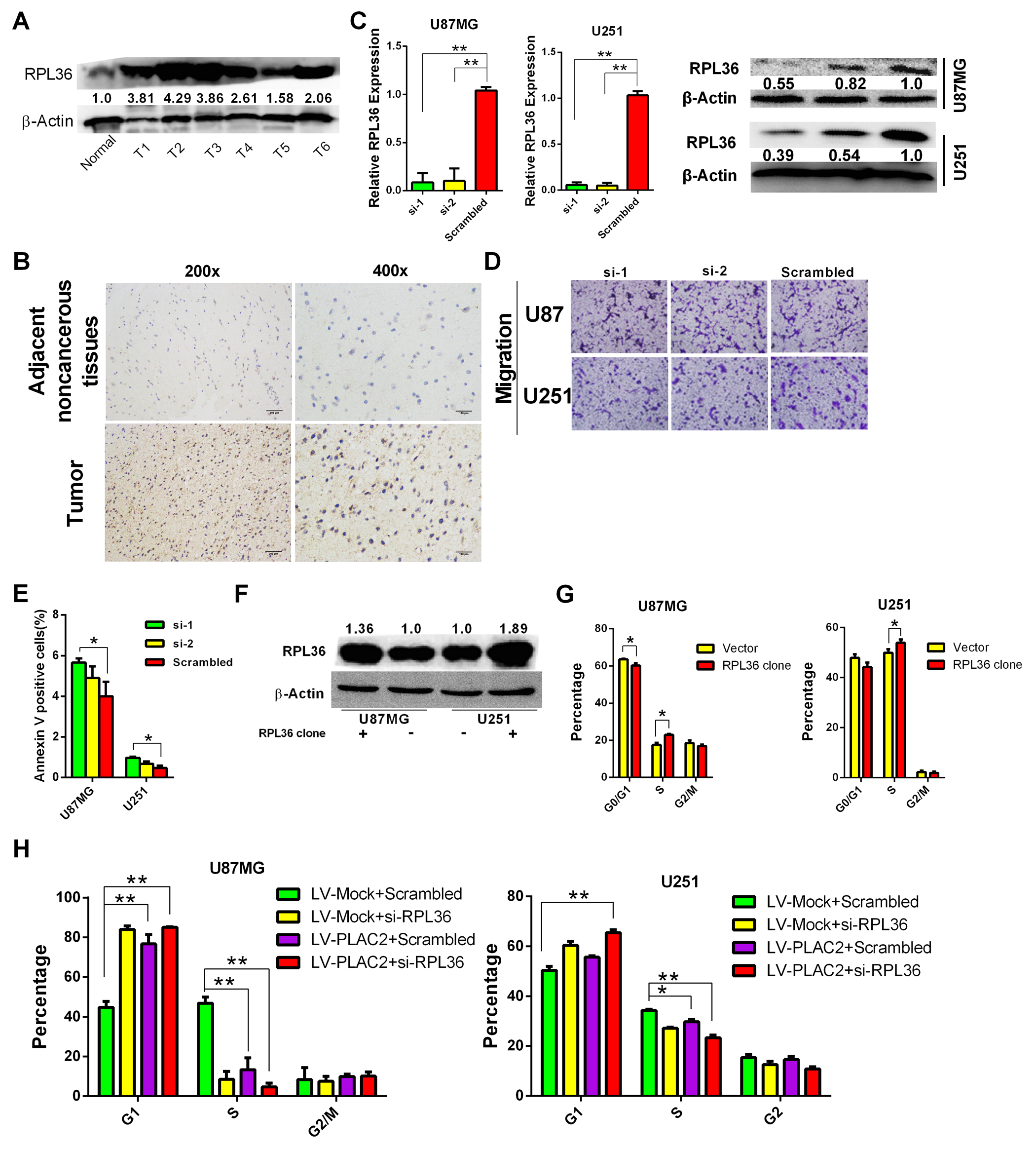

Supplement: Supplementary file 2 — Fig. S2 (A) Western blot analysis of RPL36 in glioma (T1–T6) and normal brain tissue (n = 6). Experiments were performed in triplicate. (B) Representative images of RPL36 expression in glioma (Tumor) and adjacent non‐cancerous tissue by immunohistochemistry (n = 6). (C) RPL36 mRNA and protein levels were downregulated in U87MG and U251 cells upon RPL36 knockdown by si‐1 or ‐2. Scrambled siRNA served as a negative control. (D) There was no difference in the migration of U87MG and U251 cells transfected with si‐RPL36 as compared to the respective controls, as determined by the transwell assay. (E) RPL36 knockdown increased the rate of apoptosis of U87MG and U251 cells. (F) RPL36 protein level was upregulated in U87MG and U251 cells upon transfection of plasmid expressing RPL36. *P < 0.05, **P < 0.01. (G) RPL36 overexpression induced cell cycle progression in both cell lines relative to control cells. RPL36 clone was the RPL36 expression plasmid and vector was the empty plasmid used as a control. *P < 0.05. (H) Changes in the cell cycle after cotransfection of U87MG and U251 cells with indicated treatment. RPL36 knockdown in PLAC2‐overexpressing cells enhanced G1/S arrest. [file JCMM-22-497-s002.tif]

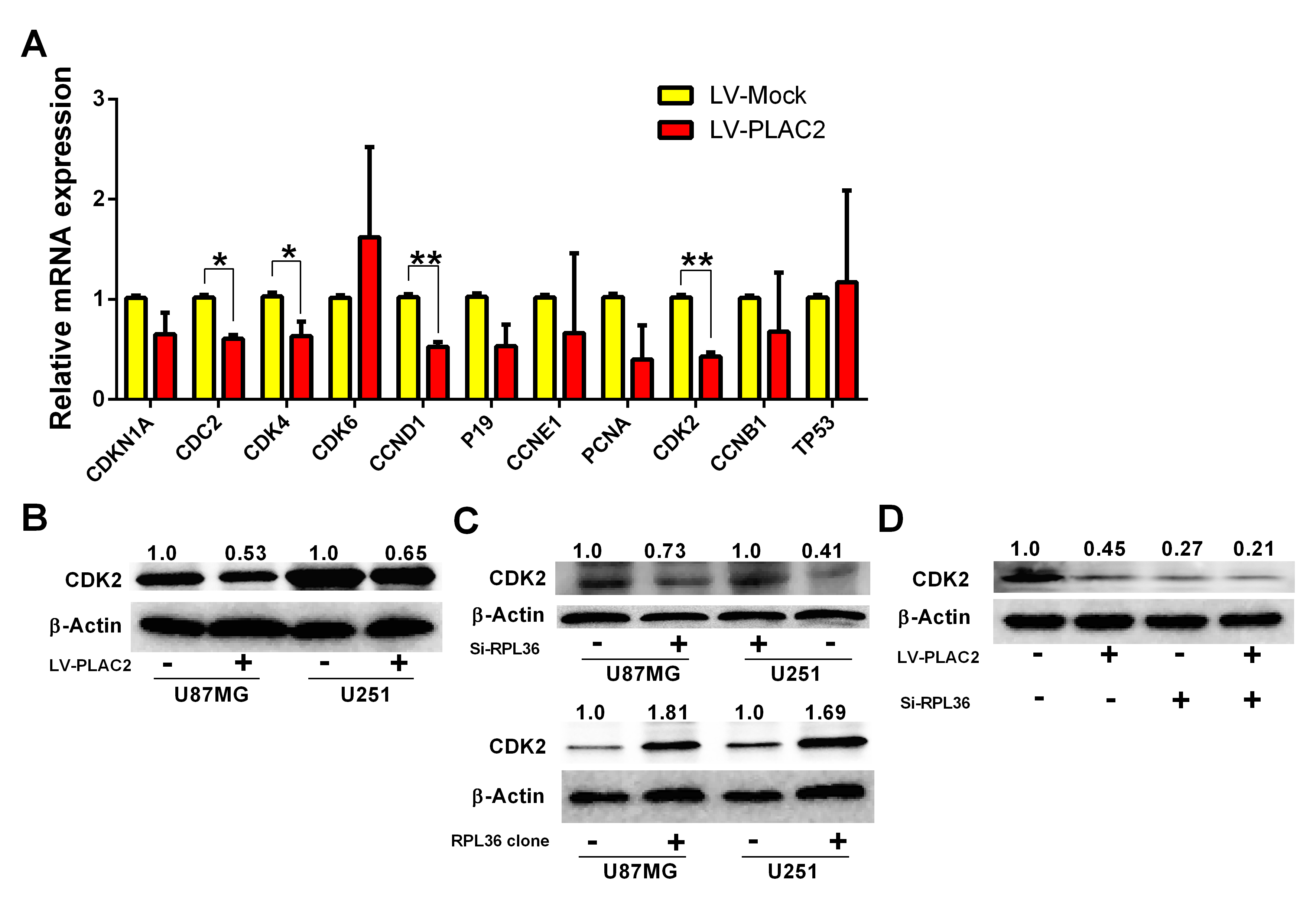

Supplement: Supplementary file 3 — Fig. S3 PLAC2/RPL36 pathway induces G1/S arrest via suppression of CDK2. (A) Relative expression levels of cell cycle‐associated genes were detected in U87MG cells overexpressing PLAC2 by qRTPCR. *P < 0.05, **P < 0.01. (B) U87MG and U251 cells were infected with LV expressing PLAC2 or an empty LV vector as a control, and CDK2 expression was evaluated by western blotting. The level was downregulated by PLAC2 overexpression. (C) CDK2 levels detected by western blotting after RPL36 knockdown or overexpression. CDK2 was upregulated in cells overexpressing RPL36 and was downregulated upon RPL36 knockdown. (D) CDK2 protein level was decreased by PLAC2 overexpression, an effect that was enhanced by RPL36 knockdown. [file JCMM-22-497-s003.tif]

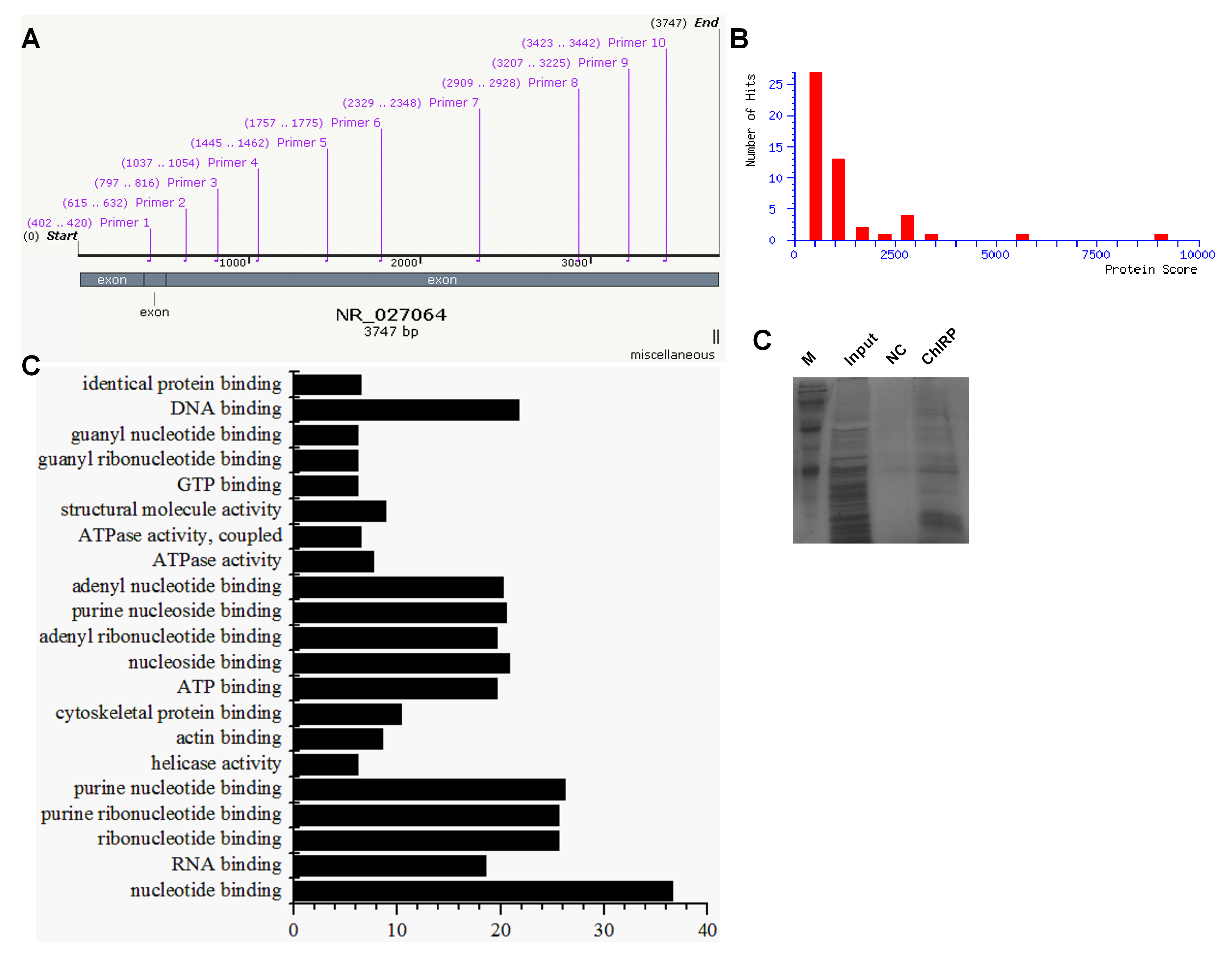

Supplement: Supplementary file 4 — Fig. S4 Schematic illustration of probes used for pulldown of PLAC2 and results of ChIRPMS. (A) Schematic illustration of the binding sites of 10 antisense DNA tiling probes on PLAC2. (B) MS data analysis performed using Matrix Science (http://www.matrixscience.com/). The number of hits represent the number of proteins obtained by ChIRP. (C) Proteins pulled down by ChIRP with a non‐targeting probe used as a control (NC), as visualized by silver staining. (D) Molecular functions or proteins identified by ChIRP‐MS according to Gene Ontology analysis. [file JCMM-22-497-s004.tif]

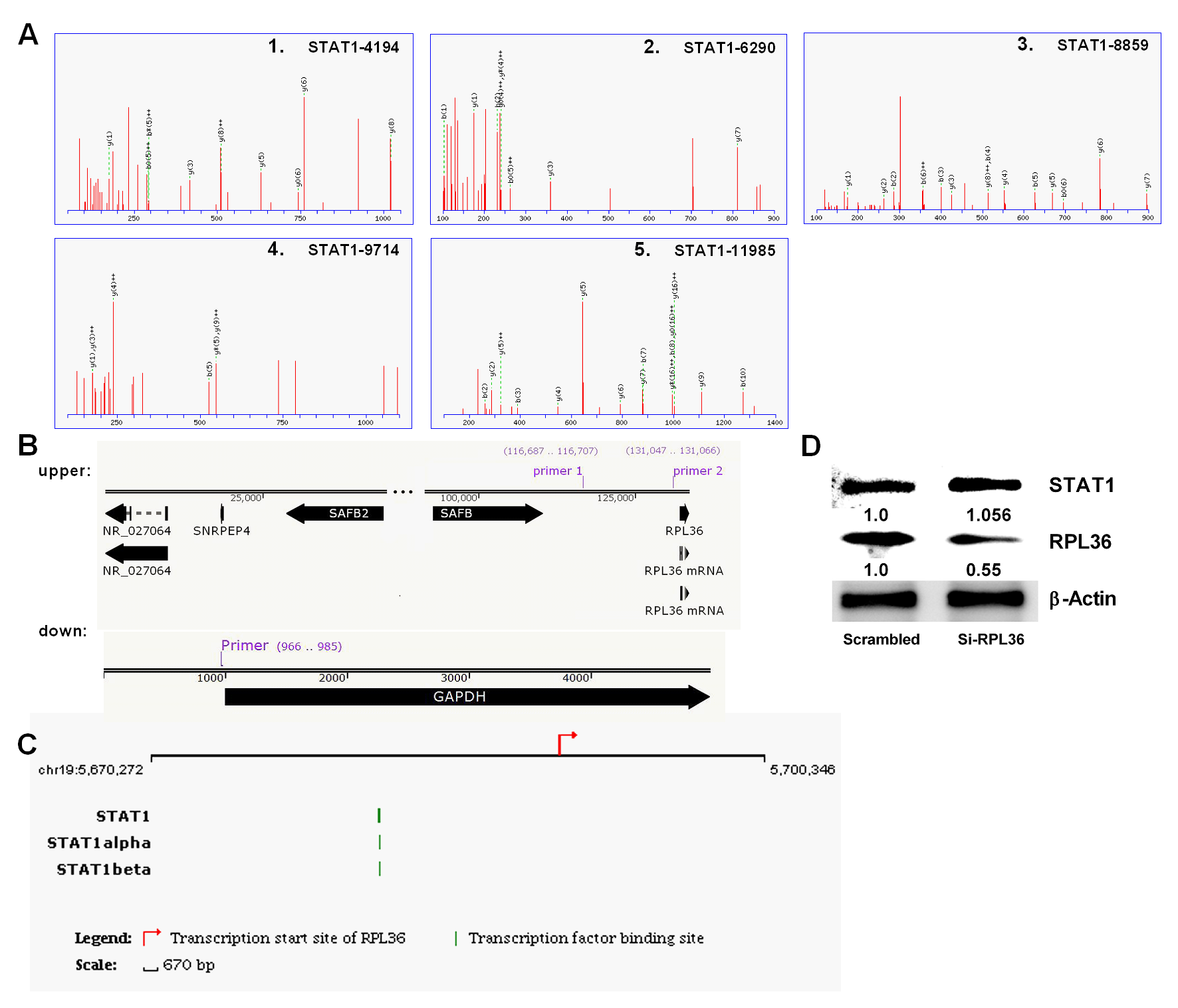

Supplement: Supplementary file 5 — Fig. S5 (A) Mass spectra of STAT1. (B) PCR primers designed with the transcription start site at −2000 to +200 bp as template amplifying a product of 100–200 bp. Schematic illustrations of primers amplifying RPL36 (above) and GAPDH (below) are shown. (C) Schematic illustration of putative STAT1 binding sites in RPL36. (D) Transfection of siRNA targeting RPL36 in U87MG cells does not have a significant effect on STAT1 expression. [file JCMM-22-497-s005.tif]
