# Supplementary material for: LncRNA PLAC2 down‐regulates RPL36 expression and blocks cell cycle progression in glioma through a mechanism involving STAT1
Source: J Cell Mol Med. 2017 Sep 18;22(1):497–510. doi: 10.1111/jcmm.13338 (PMC5742712; doi:10.1111/jcmm.13338)
Supplement: Supplementary file 6 — Table S1 Clinical and molecular pathology features of Glioma samples in association with RPL36 mRNA expression Table S2 Sequences of primers used in this study Table S3. Sequences of siRNAs used in this study Table S4 Antisense DNA tiling probes for pulldown of PLAC2 or nonsense DNA tiling probes used for ChIRP Table S5 Sequences of primers used for RIP‐PCR [file JCMM-22-497-s006.docx]

**Supplementary Table 1.** Clinical and molecular pathology features of Glioma

samples in association with RPL36 mRNA expression

|  | **Low** | **High** | **P** |
| --- | --- | --- | --- |
| Gender, female/male | 8/13 | 12/15 | .658 |
| Age at diagnosis, y | 44.6±18.4 | 34.5±19.5 | .082 |
| IDH1 mutation (no  mutation/mutation) | 7/6 | 6/8 | .568 |
| MGMT promoter  methylation  (unmethylation  / methylation) | 8/5 | 6/7 | .431 |
|  |  |  |  |
|  |  |  |  |
| Ki-67 (low/high) | 11/7 | 5/15 | .024a |

aP values less than 0.05 were considered statistically significant. All glioma samples were divided into the

RPL36 mRNA low expression group and the high expression group, and the median was used as the cutoff value.

**Supplementary Table 2.** Sequences of primers used in this study

| **Gene name** | **Forward primer,** 5ʹ-3ʹ | **Reverse primer,** 5ʹ-3ʹ | **Product size, bp** |
| --- | --- | --- | --- |
| *lncRNA PLAC2* | AATGTCTTGGCCTTGAATGA | CAAACTCAGGGATACATGGA | 120 |
| *U6* | CTCGCTTCGGCAGCACA | AACGCTTCACGAATTTGCGT | 94 |
| *RPL36* | CCTCAACAAGGGCCACAAAGT | GAATCATGTCCCGCACGAA | 104 |
| *CDKN1A* | TGTCCGTCAGAACCCATGC | AAAGTCGAAGTTCCATCGCTC | 139 |
| *CDK2* | CCAGGAGTTACTTCTATGCCTGA | TTCATCCAGGGGAGGTACAAC | 90 |
| *CDK4* | ATGGCTACCTCTCGATATGAGC | CATTGGGGACTCTCACACTCT | 124 |
| *CDK6* | GCTGACCAGCAGTACGAATG | GCACACATCAAACAACCTGACC | 225 |
| *CCND1* | GCTGCGAAGTGGAAACCATC | CCTCCTTCTGCACACATTTGAA | 135 |
| *CDKN2D* | AGTCCAGTCCATGACGCAG | ATCAGGCACGTTGACATCAGC | 87 |
| *CCNE1* | ACTCAACGTGCAAGCCTCG | GCTCAAGAAAGTGCTGATCCC | 141 |
| *PCNA* | GCGTGAACCTCACCAGTATGT | TCTTCGGCCCTTAGTGTAATGAT | 76 |
| *CDC2* | ATCCAGAACTTCGACCTTCCG | ACGGCATTGAGCGTGTAGG | 239 |
| *CCNB1* | AATAAGGCGAAGATCAACATGGC | TTTGTTACCAATGTCCCCAAGAG | 101 |
| *TP53* | CAGCACATGACGGAGGTTGT | TCATCCAAATACTCCACACGC | 125 |
| *STAT1* | CAGCTTGACTCAAAATTCCTGGA | TGAAGATTACGCTTGCTTTTCCT | 248 |
| *GAPDH* | GCACCGTCAAGGCTGAGAAC | TGGTGAAGACGCCAGTGGA | 138 |

**Supplementary Table 3.** Sequences of siRNAs used in this study

| **Gene name** | **Target sequences** | **Sense(**5ʹ-3ʹ**)** | **Antisense(3ʹ-5ʹ)** |
| --- | --- | --- | --- |
| si-h-*RPL36*  _001 | CCATGGAGTTACTGAAGGT | CCAUGGAGUUACUGAAGGU dTdT | dTdT GGUACCUCAAUGACUUCCA |
| si-h-*RPL36*  _002 | TGACCAAGAACGTGAGCAA | UGACCAAGAACGUGAGCAA dTdT | dTdT ACUGGUUCUUGCACUCGUU |
| si-h-*STAT1* | GAAAGAGCTTGACAGTAAA | GAAAGAGCTTGACAGTAAA dTdT | dTdT CUUUCUCGAACUGUCAUUU |

h, Human; si, short interfering RNA.

**Supplementary Table 4.** Antisense DNA tiling probes for pulldown of *PLAC2* or nonsense DNA tiling probes used for ChIRP

| **Probe name** | **5' - 3' sequence** |
| --- | --- |
| PC146 | AGTACCCAGGTCCCCTTGG |
| PC147 | TCCCGATAGGCTGGGCAG |
| PC148 | GGAAGTGCCTCTGAGTGTCT |
| PC149 | AGTTCGGACACGACTGGC |
| PC150 | ATGCGTAGCCTCGGCACC |
| PC151 | AGTGAAGTGGGGCTCCTCT |
| PC152 | GTCTCCATCTCCTGACCTCA |
| PC153 | GGAAGGGAAGACGGAGGAAG |
| PC154 | ACAACTCCAGCCACTGTCA |
| C1 | TCACGACGTTGTAAAACGAC |
| C2 | ATTAAGTTGGGTAACGCCAG |
| C3 | AGGTTACGTTGGTGTAGATG |
| C4 | AATGTGAGCGAGTAACAACC |
| C5 | GTAGCCAGCTTTCATCAACA |
| C6 | AATAATTCGCGTCTGGCCTT |
| C7 | AGATGAAACGCCGAGTTAAC |
| C8 | AATTCAGACGGCAAACGACT |
| C9 | TTTCTCCGGCGCGTAAAAAT |
| C10 | ATCTTCCAGATAACTGCCGT |
| C11 | AACGAGACGTCACGGAAAAT |
| C12 | GCTGATTTGTGTAGTCGGTT |

PC146 to PC154 were probes for PLAC2 pulldown; C1 to C12 were a similar set of probes targeting *LacZ* mRNA —normally absent in human cells—as the mock control.

**Supplementary Table 5.** Sequences of primers used for RIP-PCR

| **Gene** | **Primer Sequence 5ʹ-3ʹ.** | **Product, bp** |
| --- | --- | --- |
| *PLAC2* | CTGCTCCCCCAGTCTGTTTT | 246 |
|  | TCAGTGGATCCCGAGTGAGT |  |
| *GAPDH* | AACGGATTTGGTCGTATTGGG | 211 |
|  | CCTGGAAGATGGTGATGGGAT |  |
